# Assessing the Capability of Government Information Intervention and Socioeconomic Factors of Information Sharing during the COVID-19 Pandemic: A Cross-Country Study Using Big Data Analytics

**DOI:** 10.3390/bs12060190

**Published:** 2022-06-15

**Authors:** Sejung Park, Rong Wang

**Affiliations:** 1 Division of Global & Interdisciplinary Studies, Pukyong National University, Busan 48513, Korea; sjpark@pknu.ac.kr; 2 Department of Human and Organizational Development, Peabody College of Education and Human Development, Vanderbilt University, Nashville, TN 37240, USA

**Keywords:** information behaviors, COVID-19, risk management, big data analytics, social network analysis, exponential random graph modeling, online visibility

## Abstract

(1) Background: This study introduces a novel computational approach to examine government capabilities in information intervention for risk management, influential agents in a global information network, and the socioeconomic factors of information-sharing behaviors of the public across regions during the COVID-19 pandemic. (2) Methods: Citation network analysis was employed to gauge the online visibility of governmental health institutions across regions. A bipartite exponential random graph modeling (ERGM) procedure was conducted to measure network dynamics. (3) Results: COVID-19 response agencies in Europe had the highest web impact, whereas health agencies in North America had the lowest. Various stakeholders, such as businesses, non-profit organizations, governments, and educational institutions played a key role in sharing the COVID-19 response by agencies’ information given on their websites. Income inequality and GDP per capita were associated with the high online visibility of governmental health agencies. Other factors, such as population size, an aging population, death rate, and case percentage, did not contribute to the agencies’ online visibility, suggesting that demographic characteristics and health status are not predictors of sharing government resources. (4) Conclusions: A combination of citation network analysis and ERGM helps reveal information flow dynamics and understand the socioeconomic consequences of sharing the government’s COVID-19 information during the pandemic.

## 1. Introduction

Although communication plays a critical role in tackling pandemics, infodemics further accelerate the risk of COVID-19 outbreaks. Infodemic refers to the prevalence of unreliable, erroneous, or inaccurate information and is one of the biggest challenges that the world has faced [1]. Infodemics, especially the spread of unreliable information on social media, reinforced the pandemic’s power by sowing confusion, fear, and panic among the public [2]. Thus, the governments’ prompt communication about infectious diseases and the dissemination of useful information for the containment of further risk is vital during pandemics. When epidemics spread, people actively search for information to obtain knowledge about the risk of events and adopt preventive measures to protect themselves and others [3,4]. In particular, unexpected diseases like COVID-19 cause high uncertainty, resulting in feelings of helplessness, anxiety, and psychological distress [5,6]. Thus, the government’s efficient information intervention and the community outreach of public health authorities for public safety are crucial in managing outbreaks.

As the efforts of information intervention to provide up-to-date information and useful health guidelines during pandemics is imperative, public health agencies utilize their websites as major outreach platforms. People rely on information updates and guidelines from governmental public health organizations during pandemics [7]. For example, compared to other public health guidelines, adults in the United States (US) follow the Centers for Disease Control and Prevention (CDC)’s recommendations on COVID-19 fairly well [8]. However, it is questionable as to what extent such information is actually used by the various social entities on the web, what makes such risk information more viral during a pandemic, and regional variations in the impact of governments’ information intervention efforts. 

This study introduces a novel computational approach to assess the government information intervention capabilities for risk management across regions. It explores two main themes, namely: how governments’ risk information was spread on the web during the COVID-19 pandemic and which socioeconomic factors are related to information sharing behaviors of the public. To address this, we investigate the flow of risk information, the impact of government information intervention, and the factors that dictate link formation in a global information network. 

## 2. Government Information Intervention to Health Risk

Governments are responsible for risk management and resilience in health crises. In particular, unexpected diseases like COVID-19 that spread quickly necessitate more information control and timely interaction between governments and the public [9]. Government information intervention means that they disseminate up-to-date and reliable news by using their information infrastructure [9,10]. Thus, the public is able to access and utilize information to prevent the further spread of infectious diseases, adopt self-protective measures, and make informed decisions [11]. Government information-provision intervention is not only cost-effective but also an efficient means of communication to educate the public and prevent the further risk of diseases [8]. Despite these benefits, only a few studies have evaluated government information intervention effectiveness [9]. 

Previous studies have noted the advantages of successful information networks that are organized by governments. They can be useful in challenging disease risks. China’s information infrastructure, technology innovation, and the use of big data during the COVID-19 pandemic successfully managed and controlled the outbreak through epidemic trend detection [12]. In addition to executing effective information provision, key players who could generate ripple effects in information dissemination play an important role in facilitating information diffusion and meaningful interaction between stakeholders [13]. Revealing the dynamics of information networks and understanding the critical agents who spread resources and mediate others on the web help to elucidate efficient risk management [14,15]. Furthermore, it helps to identify the knowledge structure, along with interaction patterns, among users [13], information impacts [16], and the status quo in source distribution [17].

To fully utilize the potential advantages of information intervention, various stakeholders and the public should pay attention to the information and news provided by their governments and use the relevant information. One way of assessing the general public’s information-sharing behaviors related to infectious disease is to track web traffic to a country’s official central health agency. In this study, we argued that the more the public shared the government’s health-agency information related to COVID-19, the more effective its information intervention was in using its websites. Based on this discussion, this study examines the information network of governments’ health agencies who were responsible for responding to COVID-19 across countries and evaluates the web impacts of their information intervention. Therefore, we address the following research questions: 

RQ1: How did government health agencies’ COVID-19 information spread on the web?

RQ2: Who were the influential agents in the global information network regarding managing the COVID-19 pandemic?

## 3. Risk Communication and the Resilience of Infectious Disease

Risk communication can build community resilience and improve public preparedness [18]. During a pandemic, government agencies are expected to maintain regular communication to manage the uncertainty of infectious diseases by using risk communication challenges, such as by using their official websites. In a study analyzing South Korea’s 2015 Middle East Respiratory Syndrome (MERS) response, Kim et al. [19] highlighted the finding that he communication that occurs between government agencies and the public plays a significant role in risk management. 

COVID-19 carries numerous uncertainties related to its symptoms, variants, and vaccination. The literature shows that risk perception and uncertainty motivated people to seek information on COVID-19 [20]. Understanding online information-sharing highlights the implications of how people build resilience in response to a global pandemic. Resilience refers to “the ability to respond to and quickly bounce back from disruptions and stressors” [21]. 

Sharing government information increases the online visibility of the government, and in turn, can augment the capability of government information outreach to the wider public during a health crisis. Considering the fact that fake news on social media is prevalent during a pandemic, the dissemination of health authorities’ useful information is key to correcting erroneous information and providing useful knowledge on preventive measures [2,9]. The COVID-19 outbreak influenced the social, political, and economic lives of individuals and businesses. A nation’s socioeconomic factors are associated with online visibility [22]. Thus, such factors and COVID-19 status, including the number of cases, mortality, and hospitalizations, can also shape the information-sharing behavior of the public. Based on this discussion, we address the following research questions: 

RQ3: Are socioeconomic factors related to the online visibility of health government agencies during the COVID-19 pandemic?

RQ4: What social entities engaged in the public’s information-sharing behaviors in building resilience to manage the COVID-19 pandemic?

## 4. Materials and Methods

### 4.1. Data Collection

The data collection procedure took place as follows. First, we identified a list of 196 countries affected by COVID-19, based on the World Health Organization (WHO). Second, we used the DuckDuckGo search engine and the keywords “COVID-19 + Country” to locate the websites of such countries’ health agencies, which were in charge of responding to COVID-19. The search engine generated constant and unbiased results that were not influenced by users’ web history. If a country had more than one health agency tackling COVID-19, the most well-known organization appearing at the top of the search engine results was selected. This step identified 148 health agencies that had functional websites as of July 2020. Their URLs were recorded for further analysis. Third, all webpages that mentioned the URLs of such health agencies’ websites were retrieved through a bing.com search API, using Webometric Analyst 2.0 on 21 August 2020 [23]. This resulted in a total of 29,043 web pages and 148 health agencies’ websites. 

### 4.2. Citation Network Analysis 

This study demonstrates a novel analytical approach to assessing the capability of governments’ information intervention against COVID-19 for risk reduction, by revealing the impact of information in terms of URL citations on the web, across the various countries. The web impact was calculated, based on the number of URL citations a website received [23]. For example, the web pages that cited a health authority’s website URL were counted. Web-traffic volume data are commonly used to evaluate the impact of websites [24]. Compared to normalized or scaled data, such as search engine query volumes, raw web traffic data are more useful for tracking and predicting public behavior, as they reflect public interest in real time [16,25,26]. Thus, this raw web-traffic data was used to compute the network metrics.

All webpages that cited the government agencies were categorized as top-level domains (TLDs) at two levels in this study: generic top-level domains (gTLDs) and country-code top-level domains (ccTLDs). A gTLD is a proxy for the type of organization. For instance, .edu is frequently used by educational institutions, and .gov by governments (Park and Park, 2020). A ccTLD allows users to identify a domain’s geographical area. For example, .kr is generally used on websites in South Korea, and .cl on websites in Chile. A country-code second-level domain, such as .co.uk, was also coded as ccTLD.

To further investigate the information flow, a network analysis of the interconnection between TLDs and government sites was employed. In the network, nodes refer to actors connected through information-sharing and include governmental health agencies and TLDs. Edges refer to links between governmental health agencies and TLDs, while network measures such as density, centralities, average geodesic distance, and diameter were computed. UCINET and NetDraw were used for network visualization and the calculation of network descriptive attributes. 

### 4.3. Bipartite Exponential Random Graph Modeling (ERGM)

**Figure 1:**
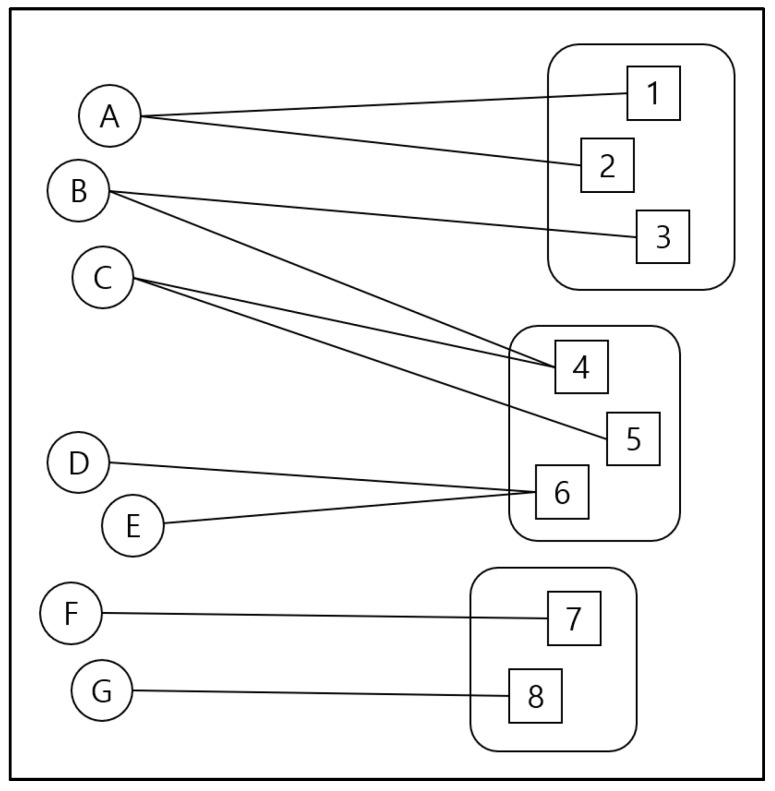
Illustrative figure to demonstrate the relationships between top-level domains and health authorities across countries. Note: This illustration was adapted from Shumate et al. [28] The existence of a tie indicates the number of web mentions from a particular top-level domain to a particular country’s official COVID-19 website. The circles with letters on the left indicate the top-level domains that cited government health agencies, while the squares with numbers on the right indicate the government health agencies’ websites. The lines between the circles and squares indicate the ties between a top-level domain and the websites of the health authorities in 148 countries, in terms of web mentions. Health authorities’ websites with the same rounded shape were in the same geographic region.

Bipartite exponential random graph modeling (ERGM) was conducted to analyze the network patterns between two sets of nodes [27] (see Figure 1 for an illustration of how the ties were coded). ERGM investigates the propensities in a network compared to variables sorted by chance alone, through simultaneously testing the effects of variables from multiple levels [28,29]. It enables the grouped analysis of health organizations across countries and TLD attributes, which reveals whether and how local parameters (at the country and TLD levels) shape the observed network configurations. 

The model fits the data seen when the *t* values of all parameters are lower [30]. A specific parameter is significant when the *t* value is within the 1.96 range of standard errors. Positive and significant coefficients in bipartite ERGM indicate that the corresponding structures are more likely to occur than random chances. 

#### 4.3.1. Country-Level Variables of Health Organizations

Country-level variables for health organizations were obtained. Three variables were used to measure a country’s inequality. First, income inequality was measured. Second, we measured the gini coefficient that captures the overall degree of inequality in a country; a larger number indicates greater inequality at the country level. Third, we used the inequality-adjusted human development index (IHDI), which combines a country’s average achievements in health, education, and income with how those achievements are distributed among the country’s population. All these variables were collected from the World Bank Development Indicator (WBDI) database. 

The death rate and case percentage were used as control variables, drawing data from John Hopkins University [31]. The population was measured using the data collected from the WBDI database. The variable was logged for analysis due to its skewness. The aging indicator was measured as the percentage of people aged 65 and above in the total population, using data collected from the WBDI database. Furthermore, we measured a country’s wealth by using its gross domestic product (GDP) per capita (in USD), which was obtained from the WBDI database.

The variable of region captures the geographic locations of each website, where 1 = Africa (*n* = 32), 2 = North America (*n* = 2), 3 = South America (*n* = 28), 4 = the Eastern Mediterranean (*n* = 17), 5 = Europe (*n* = 43), and 6 = Asia (*n* = 26). Data were coded, based on the categorization used by the WHO. 

#### 4.3.2. TLD Types

We conducted further coding of the gTLDs to identify the organizational types of TLD for each website that sent a link to government health websites. We coded all the gTLDs into four additional subcategories, including business domains (businesses in general or in general areas, including .com, .co, .global, .care, and .legal; *n* = 12), public goods domains (nonprofits, cooperatives, academic institutions, government, and the media, including .org, .or, .ngo, .ong, .net, .edu, and .gov; *n* = 16), health (*n* = 3), and others (*n* = 44). 

## 5. Results

Citation network analysis was employed to explore how government agencies’ risk reduction information on COVID-19 was spread on the web. All domains that cited the government agencies’ official websites were retrieved. Health agencies in Europe had the highest number of citations (12,195) on the web, followed by South America (7567), Africa (2989), the Eastern Mediterranean (2939), Asia (2468), and North America (885). 

**Figure 2:**
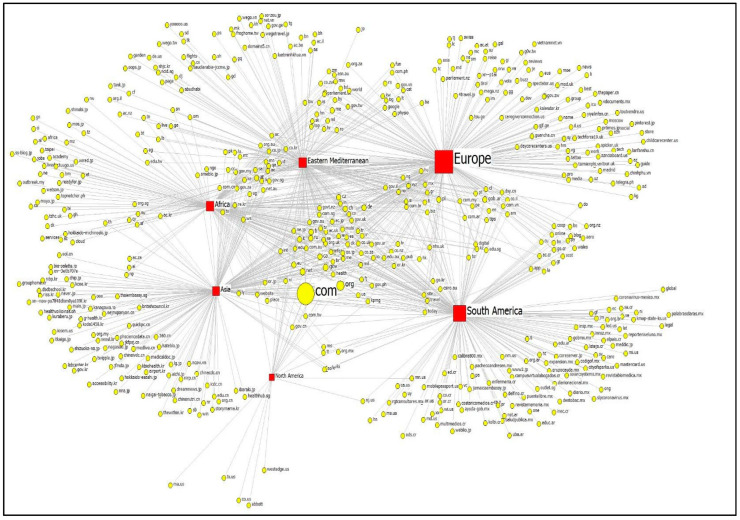
Network of COVID-19 Government Agencies across Regions and TLDs.

Figure 2 visualizes the network diagram of interconnections between the regions of government organizations and the TLDs of the websites that sent links to the websites. Square nodes refer to the regions, while circle nodes denote TLDs. A tie between government sites and TLDs means that a website with TLDs mentioned the particular government’s sites on the web. The size of a region node represents the number of links that the COVID-19 government organizations received from the TLDs. The size of a TLD node refers to the number of links that it created to the government agencies. A bigger node represents more links in the network. 

The information network of COVID-19 government agencies was sparse, with network density being relatively low at 0.004, suggesting that 0.4% of nodes in the network were connected. The average geodesic distance, which is the shortest path between any pair of nodes, was 2.916, while its diameter value was 4.000, indicating that the longest of the shortest paths between any two nodes was around four steps. 

To examine the most influential agents in a global COVID-19 information network, the network centralities of nodes (government agencies and websites that cited government agencies) were computed. Table 1 summarizes the centralities of COVID-19 government websites across regions. The COVID-19 response agencies in Europe were the most central in the information network, in terms of the degree of centralities, followed by those in South America, the Eastern Mediterranean, Asia, Africa, and North America. This means that the COVID-19-related information that was available from government agency websites in Europe was accessed on the web to a greater extent. Europe was also the highest in closeness and betweenness centralities, suggesting that the government responses and guidelines on COVID-19 that were provided by European government agencies not only spread fast but also effectively bridged other sources of information on the web.

Table 2 presents the top 30 TLDs of sources that sent links to the health organizations’ websites. Degree centrality represents the number of connections that a node has in a network [32]. TLD nodes with high degree centralities refer to sources that actively cited the government sites. 

Closeness centrality is the inverse of the sum of all the shortest paths from one node to other nodes in a network [32]. High closeness centralities mean that sources can quickly spread the information provided by government agencies through a network since they can reach other nodes more efficiently. Betweenness centrality measures the number of times that a node lies on the shortest path between other nodes in a network [32]. High betweenness centralities suggest that sources can control the information flow. The results indicate that .com, .org, .gov, and .net were the most central across all centralities in the COVID-19 information network. This can be interpreted to mean that non-profit organizations, governments, and educational websites were the major carriers and bridgers of the governments’ responses to COVID-19 and useful health information. 

### Bipartite ERGM Results

We conducted a bipartite ERGM to analyze which socioeconomic factors (e.g., equality indicators, a country’s wealth, region, population size, an aging population, the death rate, and the case percentage) reflect the impact of government health agencies’ information intervention. The results showed that several socioeconomic factors at the country level had significant effects on tie formation probability in health agencies’ information networks. This study found that health agencies in countries with higher income inequality were more likely to have high online visibility (0.01, SE = 0.006‚ *p* = 0.02). The other two inequality indicators (gini coefficient and IHDI) did not have any effect. GDP per capita, as an indicator of a country’s wealth (5.03 × 10^−6^, S.E. = 1.15 × 10^−6^), also had a positive effect. This suggests that wealthier countries have greater visibility. Health agencies in several regions showed a higher likelihood of online visibility than agencies in the reference categories of Africa: South America (0.39, SE = 0.11, *p* < 0.001), Europe (0.51, SE = 0.13, *p* < 0.001), and Asia (0.46, SE = 0.12, *p* < 0.001). Agencies in North America and the Eastern Mediterranean did not show any significant effect. Furthermore, the aforementioned factors also did not have any effect on the online visibility of the health agencies’ government websites. 

**Figure 3:**
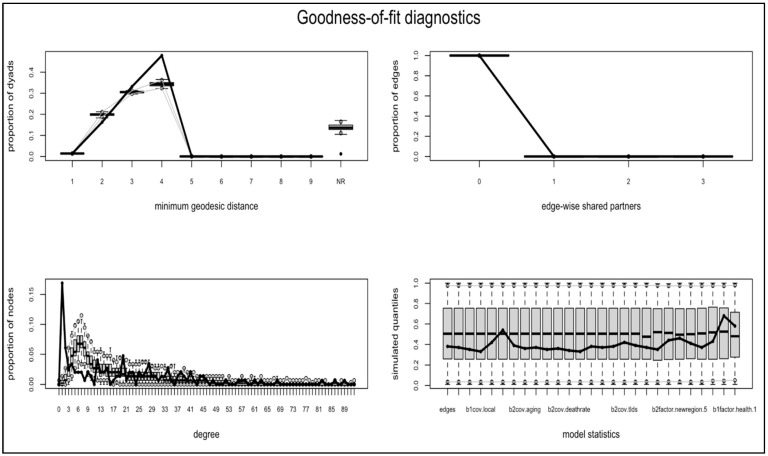
Goodness-of-fit analysis. Note: For each parameter and plotting, the vertical axis represents the log-odds of relative frequency, the statistics from the observed network are indicated by the solid lines, the boxplots indicate the median and interquartile ranges, and the light gray lines indicate the range in which 95 percent of the simulated observations fall [33]. A good fit can be concluded from the plot if the solid line primarily lies within the boxplots or gray lines.

Finally, we examined the social entities that shared information from government health agencies responsible for managing the COVID-19 pandemic. Bipartite ERGM results indicate that several domain-level attributes showed significant effects on information-sharing behavior. First, country-specific TLDs did not necessarily engage in more information-sharing, which can be explained by the global nature of the pandemic. Second, business domains were more likely to share the health government agencies’ information (0.39, S.E. = 0.20, *p* = 0.047), while public goods domains were less likely to mention the government sites (−0.33, S.E. = 0.14, *p* = 0.016). Health domains had no effect, possibly due to the small number of domains identified. Furthermore, structural parameters, such as degree centrality (43.97, S.E. = 3.80, *p* < 0.001), eigenvector centrality (−149.60, SE = 11.25, *p* < 0.001), localness centrality (644.50, S.E. = 46.55, *p* < 0.001), and betweenness centrality (−96.87, SE = 8.69, *p* < 0.001) were all significant. These findings suggest that the domains that were already locally or globally popular in the COVID-19 information network (e.g., degree centrality and localness) were more likely to be active in disseminating government agencies’ information on the web. Furthermore, domains with less influence, as measured through their connection to influential countries in the network (i.e., eigenvector centrality) or through having the shortest paths to other nodes in the network (i.e., betweenness centrality), were more connected to the government sites. The negative effects revealed here on the eigenvector and betweenness centralities indicate that some TLDs were central to information-sharing, regardless of their level of influence in the network, which motivates us to further explore the effect of TLD attributes (see the model summary in Table 3). Goodness-of-fit analysis was conducted to compare the observed graph statistics with the values of these statistics for a large number of simulated networks, based on the fitted model. The plotting showed that the model had a good fit to the data (see Figure 3). 

## 6. Discussion and Conclusions

This study demonstrates a useful analytical framework by which to assess governments’ information intervention capability in response to COVID-19 and the public’s information-sharing behaviors. This study is an initial attempt to investigate the regional differences in health authorities’ information visibility on the web and to identify the socioeconomic factors that are related to COVID-19 information spread, using big data analytics. Although previous studies have heavily relied on surveys or experiments to evaluate the effectiveness of governments’ COVID-19 responses [34,35] or to compare their approaches, we developed a new approach to revealing the extent of the health authorities’ web impact by tracking the actual information reactions of various organizations to government COVID-19 information. 

In this study, citation network analysis was used to gauge the online visibility of government health institutions. It was assumed that the more often people cited a source, the more effective that government’s information intervention is. The results indicate that COVID-19 response agencies in Europe had the highest web impact, whereas health agencies in North America had the lowest impact. Although the damage caused by COVID-19 in both regions was similarly severe in the early stages of the outbreak, both governments implemented different crisis response strategies. Europe was the most affected region as of March 2020. In particular, Italy was the most damaged country, with a high number of confirmed COVID-19 cases and a high death rate, followed by Spain, France, Germany, and the United Kingdom [36]. Although European countries’ management approaches differed, many of them employed “herd immunity” strategies and acted slowly in the early stage of the pandemic [37]. They employed strict measures later, such as limiting social contact or restricting the movements of the public. They even locked down entire countries, making stringent government interventions successful [38]. Health authorities in the US and Canada provided false information about protective gear in the early stages of the pandemic [8]. For instance, the CDC discouraged the wearing of face masks but later recommended DIY masks, which were revealed as scientifically ineffective in terms of protection. Similarly, the Public Health Agency of Canada suggested that it was not necessary for the general public to wear N-95 face masks, with the intention of reserving enough of a supply for health professionals. 

The lowest web impact seen in health agencies in North America implies a low level of trust in its public health authorities, indicating that the public health agencies’ information intervention was ineffective. This may be attributed to the lack of clarity in explaining COVID-19-related guidance and the politicization of COVID-19-related health precautions [39]. For instance, the CDC provided inconsistent guidance regarding masks and quarantine policy [40]. This result is also in line with the mistrust of the CDC that was common among social media users [41]. 

The findings also show that various stakeholders, such as businesses, non-profit organizations, governments, and educational institutions played a key role in sharing the COVID-19 response agencies’ information on their websites. This can be interpreted in the fact that they sought information regarding safe business operation, the safety of customers, and efforts to enhance resilience collectively, in order to protect other people by disseminating credible sources. They also suggest that government agencies could leverage organizational stakeholders’ urgency and their need for health information, seeking to advocate the necessary guidance. In other words, organizational stakeholders could function as information brokers between the government agencies and the general public. 

It is noteworthy that income inequality and a country’s wealth were associated with the high online visibility of governmental health agencies. This means that people living in countries with higher income gaps tended to seek government information about COVID-19 more than people who live in countries with lower income gaps. One possible explanation is that the notion of vulnerability in countries with higher income inequality may be the result of more severe cases and a higher mortality rate [42]. Therefore, people in these countries have more of a sense of urgency and a need to obtain knowledge and appropriate guidelines for protective behavior during an unexpected pandemic. Furthermore, people living in wealthier countries are more likely to seek information about COVID-19 from government sources. This might be explained by the fact that wealthier countries often have better access to technologies and health services that allow their residents to actively seek health-related information [43].

Surprisingly, other factors, such as population size, an aging population, death rate, and case percentage, did not contribute to the online visibility of the agencies, suggesting that demographic characteristics and health status are not related to the information-sharing behavior of the public. This may be attributable to the global spread of the virus, whereby everyone had the need to seek information. 

This study demonstrates a novel analytical approach to evaluating to what extent the information intervention of governmental health agencies is effective, from a social network perspective. Its findings can identify the implications for governmental health agencies regarding the status quo of the impacts of information outreach. A combination of citation network analysis and ERGM helped reveal the dynamics of information flow and understand the socioeconomic factors that are associated with sharing government COVID-19 information during the pandemic. Moreover, the network analyses at the macro level contribute to understanding geographical disparity, in terms of the capability of government information intervention to manage COVID-19. Considering that infodemics are prevalent in societies and threaten even more people than the COVID-19 virus, understanding how the public utilizes public authorities’ information sources is very important [2]. 

However, this study also has some limitations. The sample used in this study comprised web pages that cited 148 countries’ health authorities. Although this study selected the major government health authority in charge of each country’s COVID-19 management and resilience, some countries have more than one government health agency managing the response to COVID-19, which may have provided different opinions or conflicting information regarding COVID-19 to the public. Therefore, this study does not capture the entire public information-sharing network of diverse online resources offered by multiple agencies in each country. This limits the generalizability of our findings. Although government agencies also use social media platforms to provide responses to epidemics, this study sample was limited to their official websites. Thus, the results did not capture social media information-sharing behaviors. 

It should also be noted that a TLD was used as a proxy for the organizational type or geographical territory of an organization, but this may not exactly match the organizational type or country. As some websites in a particular country do not use ccTLDs, the study results do not fully capture the entire information behavior of organizations within geographical territories. The top TLDs in a global information network need to be interpreted in the context of COVID-19, which limits the generalizability of the results as the source characteristics may be contingent upon different social issues. Although the top TLDs reflect these organizations’ activities in sharing health agencies’ COVID-19-related information, they also reflect the relevance of COVID-19 in their business operations. For instance, some organizations perceiving greater urgency might have shared information more actively in their response to the pandemic. This calls for future research to investigate the critical agents in sharing government information intervention in other contexts. 

Another concern regarding web traffic is the assessment of the effects of government information intervention, which considers the number of citations that health organizations across the different regions received, in terms of the number of TLDs. However, there may be conspiracist websites among the source sites. Future studies should consider the impact of health authorities in relation to the number of conspiracist websites that disseminated COVID-19 conspiracy beliefs or health-related misinformation [44].

This study only investigated the regional differences in health agencies’ information interventions, which does not explain cross-national differences. Future studies should examine the national differences among major countries, such as Europe, the United States, and Canada, where the COVID-19 crisis was extremely severe. 

The web traffic of organizations can be associated with the communication strategies of organizations [16]. However, we did not test the relationships between the response strategies and impacts of each government’s information intervention. This calls for future research to examine the association between COVID-19 response strategies and public information behavior. 

In addition, this study only used the data collected in August 2020. Web traffic is fluid over time. As reported in the existing literature, web traffic can covary with events, such as rising case numbers and mortality rates, if researchers use single search terms to track search activities [36] or when using patterns of covariation of related search terms across a time series [45]. In addition, Rotter et al. [45] showed that some peaks of web traffic may occur with a lag after a particular government intervention (e.g., home isolation, banning mass gatherings) has been announced or is in effect. Thus, we call for future research to conduct a longitudinal analysis of networking configurations and information flow patterns, to understand the speed of information diffusion, temporal dynamics, and patterns of covariation among multiple variables across time. Finally, we acknowledge that education and literacy may influence people’s information-seeking, which variable was not accounted for in the current research because of a lack of data at the country level. Thus, we call for future research to unpack this influence within specific countries.

## Figures and Tables

**Table 1:** Centralities of COVID-19 government websites across regions.

Regions	Degree	Closeness	Betweenness
Europe	0.48031497	0.42656952	0.101202324
South America	0.39763778	0.41675794	0.087235093
Eastern Mediterranean	0.34055117	0.41024259	0.055006839
Asia	0.32874015	0.40891993	0.067508161
Africa	0.30511811	0.40630007	0.046052024
North America	0.04724409	0.37974051	0.005251393

**Table 2:** Top 30 TLDs in the COVID-19 information network, based on the types of centralities.

TLDs	Degree	Closeness	Betweenness
com	0.011811024	0.428974062	0.001684475
org	0.011811024	0.428974062	0.001684475
gov	0.011811024	0.428974062	0.001684475
net	0.011811024	0.428974062	0.001684475
de	0.00984252	0.427528083	0.001309713
it	0.00984252	0.427528083	0.001309713
ca	0.011811024	0.428974062	0.001684475
cz	0.00984252	0.427528083	0.001309713
fr	0.00984252	0.427528083	0.001309713
ch	0.00984252	0.427528083	0.001309713
ru	0.00984252	0.427528083	0.001309713
at	0.007874016	0.408042908	0.000639691
pl	0.007874016	0.412689805	0.000698267
gob.ar	0.005905512	0.40457204	0.000421699
ma	0.005905512	0.393892348	0.000243672
eu	0.011811024	0.428974062	0.001684475
cl	0.005905512	0.40457204	0.000421699
edu	0.011811024	0.428974062	0.001684475
be	0.00984252	0.427528083	0.001309713
es	0.00984252	0.427528083	0.001309713
ro	0.005905512	0.393892348	0.000243672
int	0.011811024	0.428974062	0.001684475
lt	0.003937008	0.385707051	0.00009826798
pe	0.005905512	0.40457204	0.000421699
ie	0.00984252	0.427528083	0.001309713
info	0.011811024	0.428974062	0.001684475
uy	0.001968504	0.366041362	0
gov.au	0.00984252	0.427528083	0.001309713
dk	0.00984252	0.427528083	0.001309713
pt	0.005905512	0.40457204	0.000421699

**Table 3:** Results from the bipartite ERGM.

Parameter	Estimate	S.E.	*p* Value
Edges	−7.32	0.43	<0.001
b1cov. degree	43.97	3.80	<0.001
b1cov.eigenvector_degree	−141.60	11.25	<0.001
b1cov.local	644.50	46.55	<0.001
b1cov.closeness	−0.001	0.001	0.25
b1cov.betweenness	−96.87	8.69	<0.001
b2cov.population.logged	0.01	0.01	0.31
b2cov.aging	−1.28 × 10^−5^	2.88 × 10^−4^	0.96
B2cov.GDP.per.capita	5.03 × 10^−6^	1.15 × 10^−6^	<0.001
b2cov.incomeinequality	0.01	0.006	0.02
b2cov.gini	−0.003	0.008	0.72
b2cov.ihdi	−0.25	0.33	0.44
b2cov.deathrate	−1.10	0.92	0.23
b2cov.casepercentage	1.06	3.99	0.79
b2cov.urls	0.008	0.001	<0.001
b2cov.sites	−0.001	0.001	0.27
b2cov.domains	−0.007	0.001	<0.001
b2cov.tlds	0.01	0.006	0.04
b2cov.stlds	0.03	0.005	<0.001
b2factor_North_America	−0.02	0.27	0.94
b2factor_South_America	0.39	0.11	<0.001
b2factor_Eastern Mediterranean	0.21	0.12	0.08
b2factor_Europe	0.51	0.13	<0.001
b2factor_Asia	0.46	0.12	<0.001
b1factor_cctld	0.06	0.08	0.45
b1factor_allpublic	−0.33	0.14	0.016
b1factor_business	0.39	0.20	0.047
b1factor_health	0.35	0.30	0.24
AIC = 15,504; BIC = 15,762			

## Data Availability

Not applicable.
